# From formation history to intra-articular service: coupling network evolution, fatigue, degradation, and drug release in adaptive hydrogel microspheres

**DOI:** 10.3389/fbioe.2026.1979560

**Published:** 2026-10-09

**Authors:** Yang Gu, Fude Jiao, Yuqin Fang, Jun Zhang, Yong Hu, Yunqiang Zhuang

**Affiliations:** 1 Trauma Orthopedics Center, Ningbo No. 6 Hospital, Ningbo, Zhejiang, China; 2 Ningbo Clinical Research Center for Orthopedics, Sports Medicine & Rehabilitation, Ningbo, Zhejiang, China; 3 Spine Surgery Center, Ningbo No. 6 Hospital, Ningbo, Zhejiang, China

**Keywords:** degradation, dynamic networks, fatigue, formation history, hydrogel microspheres, intra-articular delivery, mechanoresponsive drug release

## Abstract

Hydrogel microspheres intended for intra-articular delivery are commonly compared by particle size, encapsulation efficiency, equilibrium modulus, static degradation, and cumulative drug release. These endpoints do not recover the specimen’s life-cycle history. Crosslinker transport, reaction kinetics, washing, storage, reconstitution, and needle passage can leave gradients in crosslink density, water content, residual functionality, cargo distribution, and relaxation. In the joint, that hidden state evolves under compression, shear, friction, and wear, while reversible adaptation competes with defect accumulation, bond loss, degradation, and erosion. Each process can alter transport pathways and local exposure. This Mini Review examines evidence linking formation history to intra-articular service, emphasizing hydrogel microspheres rather than bulk injectable gels. Direct end-to-end evidence remains sparse: only one particle-containing study identified here compared release after repeated compression, and tribology, injection, storage, release, and pharmacokinetics are usually tested separately. We integrate findings from external gelation, dynamic networks, hydrogel fatigue, mechanically modulated release, synovial-fluid interfaces, and joint pharmacokinetics. A service-aware framework follows initially matched particles through storage, reconstitution, injection, relaxation, cyclic loading, wear, degradation, release, and repeat administration. The critical design variable is the measurable network state inherited from manufacture and altered by use, not a bond label or initial modulus alone.

## Highlights

- Formation history can persist as crosslinking, cargo-partitioning, swelling, or relaxation differences that particle size and initial modulus do not capture.
- Storage, reconstitution, and needle passage extend formation history before intra-articular service begins.
- During cyclic service, reversible adaptation competes with friction, wear, fatigue, degradation, and use-dependent release.
- Direct linked evidence remains sparse, and formulation release must remain distinct from joint retention, tissue access, and active-drug exposure.

## 1 Introduction

Hydrogels retain water-rich microenvironments, accommodate cargo, and regulate diffusion through network structure (Li and Mooney, 2016). Reviews have mapped injectable-hydrogel chemistries, gelation routes, drug classes, and translational constraints (Guo et al., 2025; Pashuck, 2026). Osteoarthritis-focused accounts organize hydrogel microspheres by cargo, targeting, lubrication, immunomodulation, and cartilage repair (Zhao et al., 2025; Zhang et al., 2026). This breadth leaves a narrower question unresolved: how does the state created during manufacture affect a microsphere as it is stored, injected, mechanically used, degraded, and dosed again?

A microsphere does not enter the joint as an ahistorical material. Its network is assembled while solutes move, bonds form, water redistributes, and cargo partitions; storage, reconstitution, and needle passage can alter that state before joint contact. Once injected, the particle may roll, compress, slide, swell, relax, erode, or become confined between tissues. Convenient assays separate these events, although they are coupled during use. Here, formation history denotes the processing-dependent network state present after fabrication and equilibration, including spatial crosslinking, relaxation spectrum, swelling tendency, cargo distribution, and susceptibility to degradation. We follow this state through pre-administration handling, cyclic service, degradation, release, and local exposure. The aim is to distinguish direct microsphere evidence from adjacent hydrogel evidence and define measurements that can test the complete chain.

## 2 Formation history as a hidden state variable

### 2.1 Formation and network development occur together

Microsphere fabrication is often represented as a change from a liquid droplet to a finished gel. External gelation shows why this endpoint view is incomplete. Multivalent ions diffuse from the bath into an alginate-rich phase, so gelation can begin at the interface while the core remains less crosslinked. Kaklamani et al. (2014) observed strong effects of ion identity and concentration on gelation time and Young’s modulus in externally gelled alginate. Although their specimens were macroscopic, the transport-reaction competition also applies to droplets: a shorter diffusion distance does not remove the moving gel front.

Particle size is therefore meaningful only relative to crosslinker transport and bond formation. Diffusion time increases with the square of transport distance, whereas reaction time depends on ion identity, concentration, polymer accessibility, and binding. Fast interfacial binding can preserve a shell-to-core difference even in a small particle. Bath volume, crosslinker depletion, residence time, and agitation belong to the material history and should be evaluated after washing and equilibration, when a transient gradient may have relaxed or remained encoded (Kaklamani et al., 2014; Cheng et al., 2011).

Cheng et al. (2011) modeled crosslinked hydrogel microspheres as spatially nonuniform matrices, combining surface-to-core crosslinking, enzymatic degradation, and an initially nonuniform small-molecule distribution. Release changed with both crosslink density and the starting cargo profile. An apparent burst can therefore reflect where cargo was located after fabrication rather than only the diffusivity of a uniform network. Encapsulation and network formation are concurrent, not independent, processes.

Processing also sets a kinetic state in dynamic covalent or reversible networks. Bond exchange can continue after a particle becomes handleable, and post-cure duration, washing, temperature, and time before testing may change the distribution of load-bearing bonds. Some effects decay during equilibration; others persist as gradients or distinct relaxation spectra. A nominal formulation is therefore insufficient when function depends on rearrangement.

### 2.2 Initial modulus does not uniquely define network state

An equilibrium or small-strain modulus compresses several network features into one number. Dynamic covalent networks can share initial stiffness while differing in bond lifetime, exchange mechanism, connectivity, and relaxation. Marco-Dufort et al. (2020) linked molecular exchange kinetics to macroscopic relaxation. Lagneau et al. (2023) used boronate-ester chemistry and precursor selection to obtain dynamic hydrogels with limited swelling and long-term stability, whereas Mihajlovic et al. (2022) combined reversible crosslinks with different lifetimes in a chondroitin sulfate/hyaluronic acid double network.

These were not intra-articular microsphere service tests, but they establish that network response is timescale dependent. A modulus measured at one frequency or rate cannot identify the relaxation spectrum governing another. Microsphere batches matched in size, cargo loading, water content, and initial modulus can therefore remain mechanically distinct; such matched comparisons reduce the chance that later differences in fatigue or release simply reflect gross stiffness or dose.

Radial composition or modulus mapping, swelling before and after equilibration, residual reactive-group assays, and multi-timescale stress relaxation can test whether processing leaves a persistent signature. When spatial measurements are impractical, relaxation and swelling provide integrated readouts but do not prove a particular gradient. Figure 1 separates experimentally supported local links from cross-stage connections that remain untested.

**FIGURE 1:**
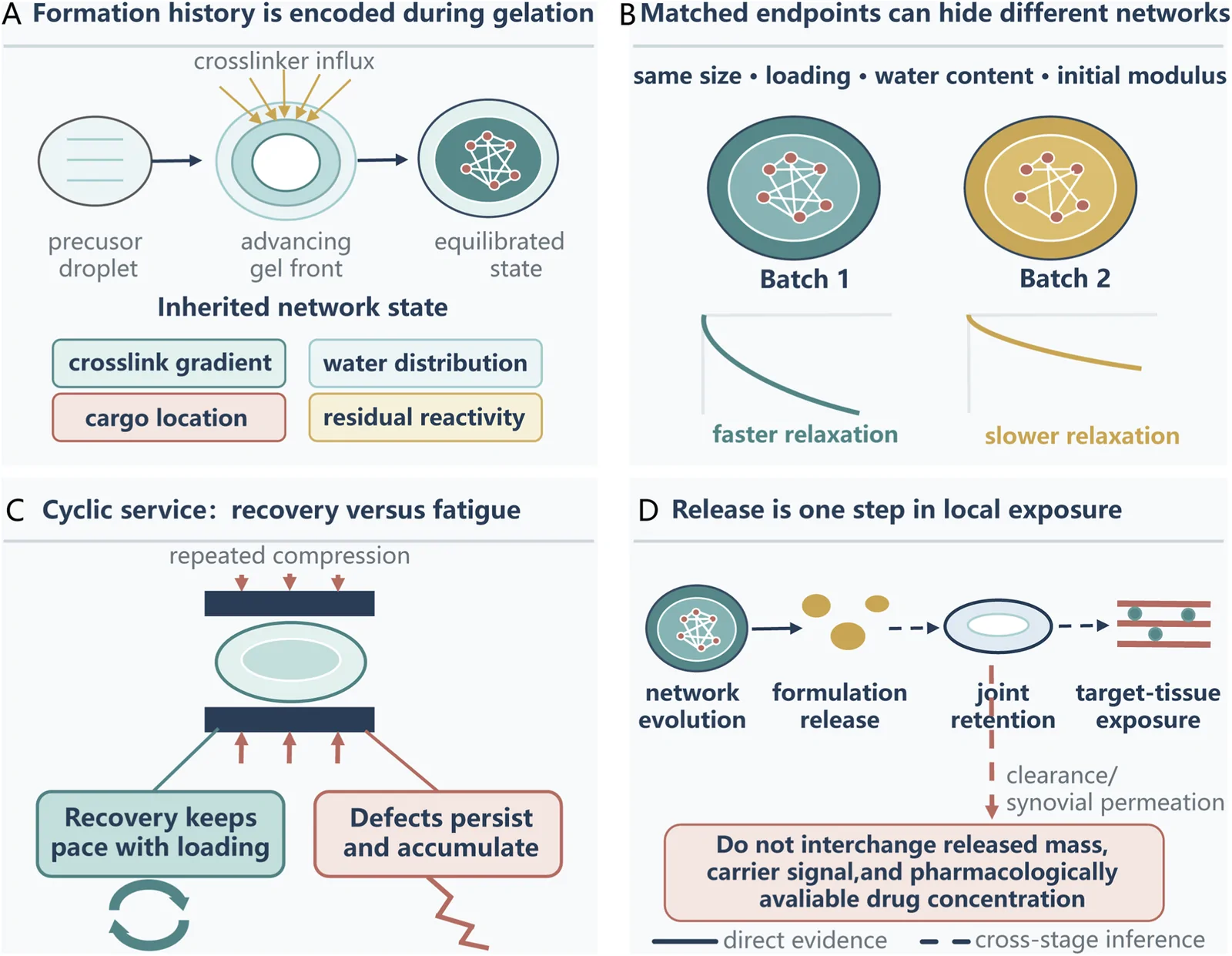
From formation history to intra-articular service. Conceptual evidence map for adaptive hydrogel microspheres. **(A)** Gelation and cargo partitioning occur over time and can create spatially nonuniform initial states. **(B)** Microspheres with similar size, loading, water content, and initial modulus may differ in crosslink distribution and relaxation spectrum. **(C)** During cyclic service, bond recovery and water redistribution compete with defect accumulation, degradation, and erosion; the outcome depends on the relation between recovery and loading timescales. **(D)** Network evolution can alter formulation release, but released mass must be distinguished from joint retention, synovial permeation, tissue uptake, and active-drug exposure. Solid arrows indicate links supported directly within individual experimental systems. Dashed arrows identify cross-stage connections that remain to be tested in an end-to-end intra-articular microsphere study.

### 2.3 Storage, reconstitution, and injection extend formation history

Formation history continues after gelation. Freezing, dehydration, lyophilization, and rehydration can alter water distribution, pore structure, aggregation, cargo retention, mechanics, and release. In alginate-based microspheres, morphological survival depended on particle architecture and freezing route (Callewaert et al., 2007). Smaniotto et al. (2020) found that particle-size distributions could recover after freeze-drying and rehydration while a microparticle-forming formulation remained rheologically altered after 48 h and showed a lower fitted nicotinamide release constant. These related systems were not drug-loaded intra-articular microspheres and used single processing cycles. They nevertheless show that restored diameter does not establish recovery of network state or release. Protectant composition, freezing and drying conditions, storage time, number of freeze-thaw cycles, and rehydration procedure should be reported with pre/post particle, mechanical, cargo-retention, and release measurements.

Needle passage adds converging flow at the entrance, transient shear and extension, wall contact, and particle-particle compression. Relevant variables include needle gauge and length, syringe geometry, injection rate, particle concentration, particle-to-bore size ratio, deformability, and carrier viscosity. Existing studies provide complementary endpoints: Stealey et al. (2025) measured 27-gauge extrusion force; Palmese et al. (2024) reported preserved size and morphology and a preliminary macromolecular-release comparison before and after 27-gauge passage; and Dharmesh et al. (2023) found viscosity recovery after a high-shear history and no qualitative morphological change after 27-gauge passage. None directly measured internal particle stress or the complete post-injection network state. Successful extrusion, acceptable force, or estimated wall shear should therefore be paired with pre/post size, morphology, cargo retention, network-sensitive mechanics, and release.

## 3 Network evolution during intra-articular service

### 3.1 Joint loading supplies boundary conditions, not a particle stress value

Instrumented knee implants record activity-dependent joint forces during walking, stair negotiation, and daily tasks (Kutzner et al., 2010; Bergmann et al., 2014). These data justify cyclic compression and shear as boundary conditions but do not specify stress on a free microsphere. Local loading depends on particle concentration and mobility, position, contact geometry, tissue clearance, and synovial rheology. Whole-joint force should not be assigned directly as microsphere stress.

Reported tribology protocols are not interchangeable. Han et al. (2021) used reciprocating contact between a polytetrafluoroethylene pin and silicon wafer at 12 N, 4-mm stroke, 1 Hz, and 600 cycles, and reported friction without a wear readout. Lei et al. (2022) used a polyethylene sphere against stainless steel at 1 N, 4 mm, 1 Hz, and 3,600 s, pairing friction with post-test particle microscopy and qualitative counterface wear tracks. Stealey et al. (2025) distinguished static and dynamic coefficients in simulated healthy and osteoarthritic synovial fluids but reported no wear endpoint. These studies establish lubrication under their test conditions, not equivalent long-term wear or fatigue resistance.

A microsphere-relevant friction protocol should report counterface materials, contact geometry, load or contact pressure, sliding or rolling mode, stroke, speed or frequency, particle concentration and configuration, medium rheology, temperature, duration, and cycle count. Wear requires longer testing with pre/post particle size and morphology, fragmentation or mass loss, debris, counterface wear tracks, surface-layer integrity, and any accompanying change in release. A low short-term coefficient of friction cannot establish progressive wear resistance.

### 3.2 Adaptation and fatigue compete over time

Repeated deformation can let reversible bonds redistribute stress, close defects, or rebuild connectivity, while the same cycles accumulate damage. Bai et al. (2017) showed that tough hydrogels can resist a severe single load yet undergo fatigue crack growth. In amorphous hydrogels with dynamic covalent bonds, fatigue resistance depended on the relation between bond recovery and deformation timescales: rapid recovery preserved connectivity, whereas slow recovery left defects available for growth (Xiao et al., 2022). Dynamic bonding is therefore not synonymous with fatigue resistance.

A lower force in later cycles can reflect recoverable solvent redistribution, bond exchange that preserves connectivity, or persistent chain scission and crack growth. Force traces alone cannot separate these routes. Loading blocks followed by defined rest intervals can estimate recoverable and persistent components, but microscopy, mass loss, or size distributions are needed before persistent softening is assigned to fatigue damage (Bai et al., 2017; Xiao et al., 2022).

Cartilage-oriented systems have begun to address durability. Yang et al. (2023) reported a cell-free hydrogel that retained function through 28,000 loading-unloading cycles and provided staged release. Zhu et al. (2026) reported stress retention after 100 cycles at 50% strain in a microsphere-embedded dual-network hydrogel, together with sustained release and chondrogenic outcomes. However, mechanics and release were tested as separate evidence streams, so neither study shows how release changed during fatigue.

Microspheres add another scale: a particle can deform internally, slide or roll, rearrange within a packed bed, or move away from loading. Granular hydrogels with dynamic interparticle crosslinks show that rearrangement can occur within particles and at particle-particle interfaces (Lee et al., 2023), but their service setting differs from a joint suspension. Experiments should state whether they test isolated particles, a packed layer, particles embedded in another gel, or a bulk analogue; otherwise, interparticle recovery can be mistaken for repair of the microsphere network.

Loading period, rest interval, amplitude, confinement, and recovery time should be reported alongside bond relaxation. Recovery after rest helps distinguish adaptation from persistent loss, while imaging or particle-size distributions can reveal fracture and erosion missed by a bulk force trace.

## 4 Coupled degradation, transport, and release

### 4.1 Direct cyclic-compression evidence and altered transport pathways

Among the studies reviewed, Li et al. (2011) provides the clearest particle-containing cyclic-load/release experiment. Poly-L-lysine-coated vascular endothelial growth factor (VEGF)/alginate microspheres, 300–400 µm in diameter, were embedded in demineralized bone matrix. The composites underwent six 1-h blocks of periodic compression, separated by 30-min intervals, at 80 cycles min−1 and 1.5-mm displacement; this daily program was repeated for 18 days. Cumulative VEGF release was 19.6% ± 1.2% after day 1 in the loaded group and 18.2% ± 1.4% without loading. By day 9, the corresponding values were 71.0% ± 1.9% and 68.5% ± 2.1%, with differences reaching significance only from days 5–9. The curves converged after day 11 and plateaued around day 15. Released VEGF retained activity toward human umbilical vein endothelial cells. The experiment directly links repeated loading history to subsequent microsphere-derived release, yet its boundary is important: the particles were constrained within a bone-matrix composite, and supernatants were sampled after the loading block and a 24-h incubation. Release pulses within individual compression cycles were not resolved.

At bulk-hydrogel scale, Sun et al. (2025) quantified kartogenin after 40 compression-rebound cycles at 40%, 50%, or 60% deformation and found greater release at greater deformation. Fang et al. (2020) reported cycle- and strain-dependent release from a tough zwitterionic hydrogel, and Oyarzún et al. (2024) found that shear rate altered both viscoelasticity and metoclopramide release from a boronic-acid-functionalized alginate/poly (vinyl alcohol) hydrogel. These studies establish load-transport coupling but differ from freely mobile intra-articular microspheres. Systems reporting durability and sustained release in parallel protocols form a separate evidence tier because they do not show that fatigue history altered release.

Deformation can change mesh dimensions and drive solvent exchange; defects can shorten diffusion paths; and erosion can liberate immobilized cargo. Their signatures differ. A compression-synchronized pulse is consistent with solvent exchange, whereas release remaining elevated after loading is more compatible with persistent permeability change, damage, or degradation. These assignments remain conditional because reversible mesh changes can outlast loading and limited erosion can escape gravimetric detection. Release should therefore be paired with mass loss, swelling, structural imaging, and recovery on the same or tightly matched samples (Tanimoto et al., 2023). Li et al. (2011) also cautions against a constant loading multiplier: loaded and unloaded curves converged as degradation advanced.

### 4.2 Release from the formulation is not equivalent to joint exposure

The surrounding medium is part of the release and mechanical system. Proteins, hyaluronic acid, and disease-relevant synovial-fluid composition altered release from biodegradable microspheres (Magri et al., 2019). Mertz et al. (2019) developed a transport-focused release model, whereas Smith et al. (2014) formulated a synovial-fluid analogue with biorelevant rheology for wear testing. A release medium must reproduce relevant solute interactions and sink conditions; a mechanical medium must also reproduce shear-dependent rheology. One formulation may not satisfy both purposes without validation.

Synovial fluid also conditions the particle surface through selective protein adsorption. The resulting interfacial layer may depend on surface chemistry, hydration, charge, network architecture, porosity, and fluid composition, and may alter aggregation, lubrication, cargo partitioning, cell interaction, or clearance. Wen et al. (2026) showed that porosity changed both the amount and composition of proteins adsorbed by rigid silica nanoparticles in healthy ovine synovial fluid and modified macrophage uptake. This adjacent-scale evidence does not establish the same behavior in soft hydrogel microspheres. Direct studies should quantify total adsorbed protein and, where feasible, composition, then relate these data to surface properties, aggregation, friction, tissue or cellular interaction, and release.

After cargo leaves a microsphere, synovial transport and clearance determine local exposure (Siefen et al., 2022). Inflamed synovium increased *ex vivo* transsynovial permeation relative to naive tissue under tested conditions (Siefen et al., 2021). Fortin et al. (2025) resolved rapid cartilage penetration and prolonged joint signal for ultrasmall fluorescent silica nanoparticles, illustrating that residence and tissue access can be separated across scales. Carrier fluorescence, however, is not intact therapeutic concentration.

Cargo released from the formulation, carrier or cargo retained in the joint, and pharmacologically available concentration at target tissue should therefore remain separate. Mechanically accelerated *in vitro* release may increase early exposure, shorten the dosing window, or have little effect when clearance dominates; a release curve alone cannot distinguish these outcomes.

## 5 A minimum service-aware test framework

A service-aware study need not reproduce every feature of joint mechanics; it should preserve causal order and measure enough state variables to explain altered performance. Table 1 follows one material lot from fabrication and equilibration through storage, reconstitution, injection, tribology, degradation, release, synovial interfacial conditioning, and repeat administration. Initially matched particles should share size, cargo loading, water content, and modulus while relaxation remains independently measured.

**TABLE 1:** Minimum evidence and reporting variables for service-aware intra-articular hydrogel microspheres.

Stage	Minimum measurements	Variables that should be controlled or reported	Interpretation boundary
Formation and equilibration	Particle-size distribution; cargo loading and spatial distribution where feasible; water content; swelling; initial modulus; multi-timescale stress relaxation	Precursor concentration and viscosity; mixing order; crosslinker concentration; droplet residence time; post-cure, washing, and equilibration history	Similar modulus does not establish a similar relaxation spectrum or radial network state
Storage and reconstitution	Pre/post size and morphology; aggregation; water and cargo retention; swelling; network-sensitive mechanics; release	Cryo-/lyoprotectant; freezing and drying protocol; storage temperature and duration; number of freeze-thaw cycles; rehydration medium, volume, time, and mixing	Recovered diameter or appearance does not establish recovery of network state or release behavior
Needle passage	Injection force or pressure; pre/post size, morphology, cargo retention, network-sensitive mechanics, and release	Needle gauge and length; syringe geometry; flow rate; particle size and concentration; particle-to-bore ratio; carrier viscosity; temperature	Successful extrusion or estimated wall shear does not establish the internal stress or integrity of an individual particle
Mechanical service and tribology	Peak and equilibrium force or stress; hysteresis; recovery; start-up and steady friction; pre/post size and morphology; fragmentation, mass loss, debris, wear tracks, and release	Particle configuration; counterfaces and contact geometry; load or pressure; waveform; sliding/rolling mode; stroke; speed/frequency; cycles; rest; medium rheology; temperature	Whole-joint force is a boundary condition; short-term low friction does not establish long-term wear or fatigue resistance
Degradation and structural evolution	Mass loss; swelling; soluble polymer or degradation products; morphology; modulus and relaxation at matched time points	Enzyme identity and activity; pH; protein and hyaluronic-acid content; medium replacement	Softening can arise from reversible relaxation, solvent redistribution, cleavage, erosion, or fracture
Release during service	Released mass before, during, and immediately after defined loading blocks; burst fraction; mass balance; sink assessment	Loading and sampling synchronized; unloaded time-matched control; medium volume and composition; agitation; replacement schedule; assay stability	Block-averaged cumulative release cannot resolve an intra-cycle pulse or identify squeezing, diffusion, damage, or degradation
Synovial interface and exposure bridge	Adsorbed protein mass and composition; surface properties and aggregation; friction; synovial permeation; retention; tissue/cell uptake; active-drug concentration	Defined or patient-derived synovial fluid; disease state; exposure time; free cargo versus carrier label; intact versus degraded carrier	Nanoparticle corona evidence is adjacent-scale; formulation release, residence, and pharmacologically available exposure are distinct
Repeat administration and batch equivalence	Residual carrier or products; response after each dose; critical material attributes and release across lots	Dose per injection; interval; cumulative dose; residual burden; lot identity; manufacturing and storage history	Single-dose or single-batch performance does not establish equivalence during repeated administration

Loading and sampling should overlap in time, with unloaded controls and defined recovery intervals. Particle size, morphology, swelling, mass loss, mechanics, and release should be measured at matched points. This design helps distinguish reversible relaxation or squeezing from persistent softening, fragmentation, and erosion without assigning mechanism from cumulative release alone.

A two-axis design keeps the framework manageable: vary formation history among equilibrated matched batches, then vary service history within one batch. The final bridge should compare formulation release with synovial permeation, joint retention, tissue uptake, or active-drug concentration. A small linked dataset is more informative than biological endpoints added to a mechanically uncharacterized formulation.

## 6 Discussion and outlook

Formation history connects manufacturing with service but raises a strict burden of proof. External gelation does not establish a radial gradient in every particle, and relaxation does not identify bond distribution. Faster exchange may dissipate stress yet also promote creep, swelling, erosion, or transport; slower exchange may retain shape while defects persist. These outcomes depend on exchange, loading, degradation, and observation timescales (Marco-Dufort et al., 2020; Lagneau et al., 2023; Xiao et al., 2022; Pashuck, 2026).

The literature contains strong local links but weak continuity across the chain. External gelation and release modeling connect processing with initial structure; dynamic-network and fatigue studies link kinetics to relaxation and recovery; mechanoresponsive hydrogels show deformation-dependent release; and intra-articular models show that medium and synovial transport reshape exposure. Li et al. (2011) remains the only particle-containing experiment identified here that directly compared periodically loaded and unloaded release. No study resolves release from freely mobile hydrogel microspheres during individual compression and rest intervals while tracking recovery or damage. Storage, injection, tribology, release, and pharmacokinetics also remain mostly disconnected protocols.

Repeat administration begins in a potentially non-naive joint. Residual carrier or degradation products, prior tissue responses, and changed synovial composition may alter confinement, protein adsorption, degradation, clearance, and exposure from a later dose. Studies should report dose per injection, interval, cumulative dose, measurable residual carrier burden, and whether doses came from the same lot. If lots or manufacturing conditions differ, equivalence should be assessed using size distribution, cargo loading, water content, swelling, relaxation or other network-sensitive mechanics, degradation, and release. Single-dose, single-batch results should not be extrapolated automatically to repeated administration.

The central experiment compares particles that look similar after equilibration but remember fabrication differently. If matched batches diverge after service and that divergence tracks relaxation, structural change, and release, formation history becomes useful; null results are equally informative. Configuration, disease-dependent fluid and enzymes, and exposure history remain part of material definition. The decisive question is which feature of the formed network remains measurable during use and reproducibly alters local drug exposure.
